# ADAMTS1 induces epithelial-mesenchymal transition pathway in non-small cell lung cancer by regulating TGF-β

**DOI:** 10.18632/aging.204594

**Published:** 2023-03-20

**Authors:** Xueqian Hu, Chunqi Jiang, Ning Hu, Shanyi Hong

**Affiliations:** 1 Department of Oncology, Ningbo Municipal Hospital of TCM, Affiliated Hospital of Zhejiang Chinese Medical University, Ningbo, China; 2 Department of Cardiovascular Division, Ningbo Municipal Hospital of TCM, Affiliated Hospital of Zhejiang Chinese Medical University, Ningbo, China; 3 Department of Internal Medicine, Ningbo Municipal Hospital of TCM, Affiliated Hospital of Zhejiang Chinese Medical University, Ningbo, China

**Keywords:** non-small cell lung cancer, ADAMTS1, TGF-β, EMT, pathway

## Abstract

Non-small cell lung cancer (NSCLC) accounts for approximately 80% of all lung cancers. Identifying key molecular targets related to the initiation, development, and metastasis of lung cancer is important for its diagnosis and target therapy. The ADAMTS families of multidomain extracellular protease enzymes have been reported to be involved in many physiological processes. In this study, we found that ADAMTS1 was highly expressed in NSCLC tissues, which promoted cell proliferation, migration, invasion, and epithelial to mesenchymal transition (EMT) of NSCLC cells. In the NSCLC tumor metastasis model involving nude mice, overexpression of ADAMTS1 promoted EMT and lung metastasis of tumor cells. Moreover, ADAMTS1 positively regulated TGF-β expression, and TGF-β was highly expressed in NSCLC tumor tissues. si-TGF-β or inhibition of TGF-β expression through the short peptide KTFR on ADAMTS1 protein could reverse the oncogenic effects of ADAMTS1 on lung cancer cells. Taken together, ADAMTS1 functioned as an oncogene in NSCLC cells by promoting TGF-β expression, indicating that ADAMTS1 has important regulatory roles in the progression of NSCLC.

## INTRODUCTION

Lung carcinoma is a malignant tumor originating in the bronchial mucosa or glands [1]. According to histopathological classification, lung carcinomas can be divided into non-small cell lung cancer (NSCLC) and small cell lung cancer (SCLC), among which NSCLC is the more common, accounting for approximately 80% of all lung carcinoma cases [2, 3]. Tumor metastasis is a complex pathological process, regulated by different genes and proteins at every stage of development [4, 5]. Therefore, exploring the mechanism of NSCLC metastasis and the interaction between invasive genes and proteins make much sense for its target therapy.

Epithelial-mesenchymal transition (EMT) is the initial and key step of metastasis in NSCLC cells [6, 7]. EMT-produced cells can undergo certain morphological changes, acquire strong migration and invasion capabilities, and have an anti-apoptotic capability and a capacity to degrade the extracellular matrix [8]. EMT mainly occurs in many physiological and pathological processes, such as tissue and embryo development, organ damage repair, organ fibrosis, and metastasis of malignant tumors, and involves complex signaling pathways [9–11]. The main molecular characteristics of EMT have been found to comprise decreased expression of E-cadherin, occludin, and cytokeratin, with increased expression and function of N-cadherin, vimentin, fibronectin, and other interstitial cell markers [12, 13].

A disintegrin and metalloproteinase with thrombospondin motifs (ADAMTS), a family that involves a depolymerized protein-like metalloproteinase containing TSP, is a new type of Zn^2+^ dependent secretory metalloproteinase identified after matrix metalloproteinases (MMPs), which is widely present in mammals and invertebrates [14]. The ADAMTS family comprises disaggregated protein-like metalloproteinases (ADAMs) and MMPs belonging to the metalloproteinase family. However, they differ greatly in substrate specificity, tissue distribution, and *in vivo* inhibitors [15]. Currently, the understanding of ADAMTS remains considerably unclear. The ADAMTS family of multidomain extracellular protease enzymes has been reported to be involved in many physiological processes, such as collagen maturation [16], mucin degradation [17], inhibition of angiogenesis [18], regeneration [19], and inflammation [20]. Recently, it has been found that ADAMTS plays a significant role in tumor initiation, development, and metastasis by directly affecting cell proliferation, apoptosis, migration, invasion, and angiogenesis [21, 22]. However, few studies have reported the role of ADAMTS1 in NSCLC. Therefore, this study aimed to observe the regulatory effect and mechanism of ADAMTS1 on the proliferation, cell cycle, migration, and invasion of NSCLC cells and on EMT.

## RESULTS

### ADAMTS1 is up-regulated in non-small cell lung cancer tumor tissues

**Figure 1:**
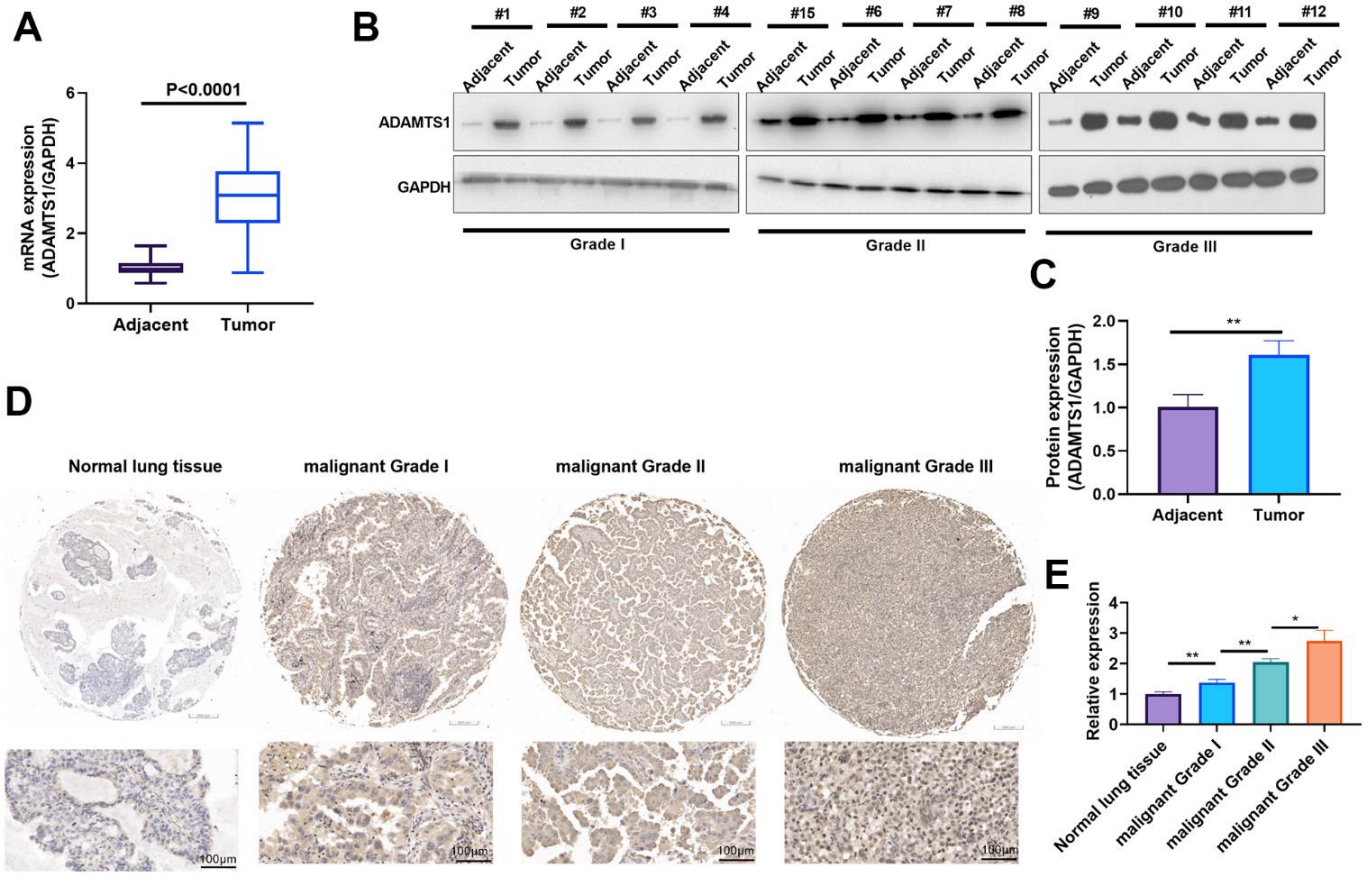
**ADAMTS1 is up-regulated in non-small cell lung cancer tumor tissues.** (**A**) qRT-PCR was conducted the mRNA expression of ADAMTS1 in NSCLC tumor tissues than that in adjacent normal tissues. (**B**, **C**) Western blot was used for evaluating the protein expression of ADAMTS1 in NSCLC tumor tissues and adjacent normal tissues with different clinical grades (n=12). (**D**, **E**) The expression of ADAMTS1 was detected in NSCLC tissues and adjacent normal tissues, (scale bar=100 μm). *P<0.05, **P<0.01.

To evaluate ADAMTS1 expression in NSCLC, qRT-PCR and Western blot were respectively used to detect ADAMTS1 mRNA and protein levels in NSCLC tumor tissues and adjacent normal tissues. The results showed that the mRNA and protein expressions of ADAMTS1 in tumor tissues were significantly higher than those in adjacent normal tissues (Figure 1A–1C). The expression of ADAMTS1 was detected in NSCLC tissues and normal tissues using immunohistochemistry, which showed that the positive signal of ADAMTS1 in tumor tissues was significantly higher than that in adjacent normal tissues (Figure 1D, 1E). These results indicate that enhanced ADAMTS1 expression may be correlated with NSCLC.

### ADAMTS1 promotes migration, invasion, and EMT in NSCLC cells and human normal lung epithelial cells

**Figure 2:**
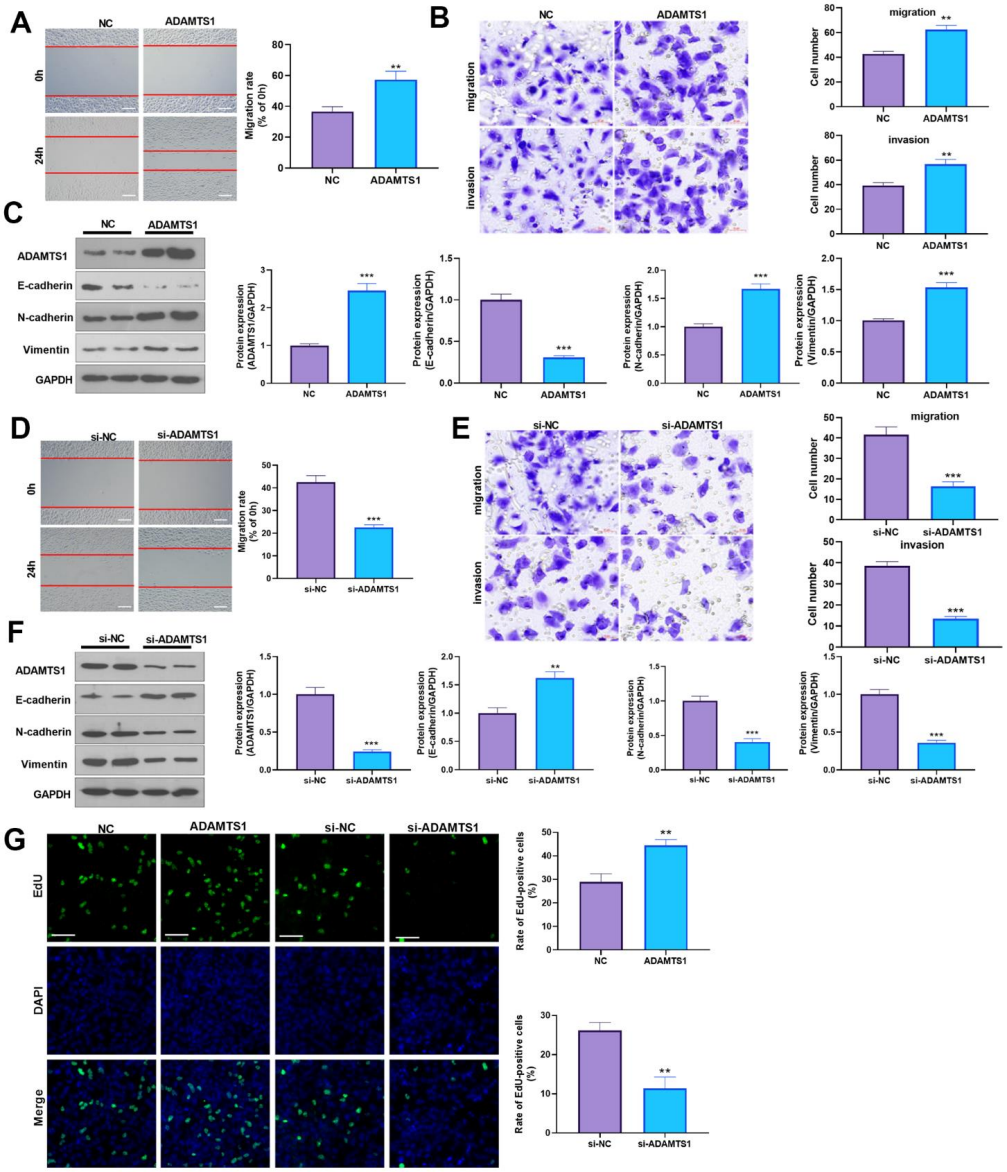
**ADAMTS1 promotes migration, invasion, and EMT of A549 cells.** ADAMTS1 overexpression plasmids or si-ADAMTS1 were transfected into A549 cells. (**A**) Wound-healing assay was used for detecting the migration of A549 cells transfected with ADAMTS1 overexpression plasmids. (**B**) Transwell examined the migration and invasion of A549 cells (scale bar=50 μm). (**C**) Protein expressions of E-cadherin, N-cadherin, and Vimentin in A549 cells were detected using Western blot. NC=negative control. (**D**) The wound-healing assay showed the migration of A549 cells after the knockdown of ADATMS1. (**E**) Transwell assays showed the migration and invasion of A549 cells after the knockdown of ADATMS1 (scale bar=50 μm). (**F**) Protein expressions of E-cadherin, N-cadherin, and Vimentin in A549 cells were detected using Western blot. (**G**) EdU staining was used for detecting cell proliferation. Scale bar=100 μm. **P<0.01, ***P<0.001 compared with NC or si-NC group. N=3.

**Figure 3:**
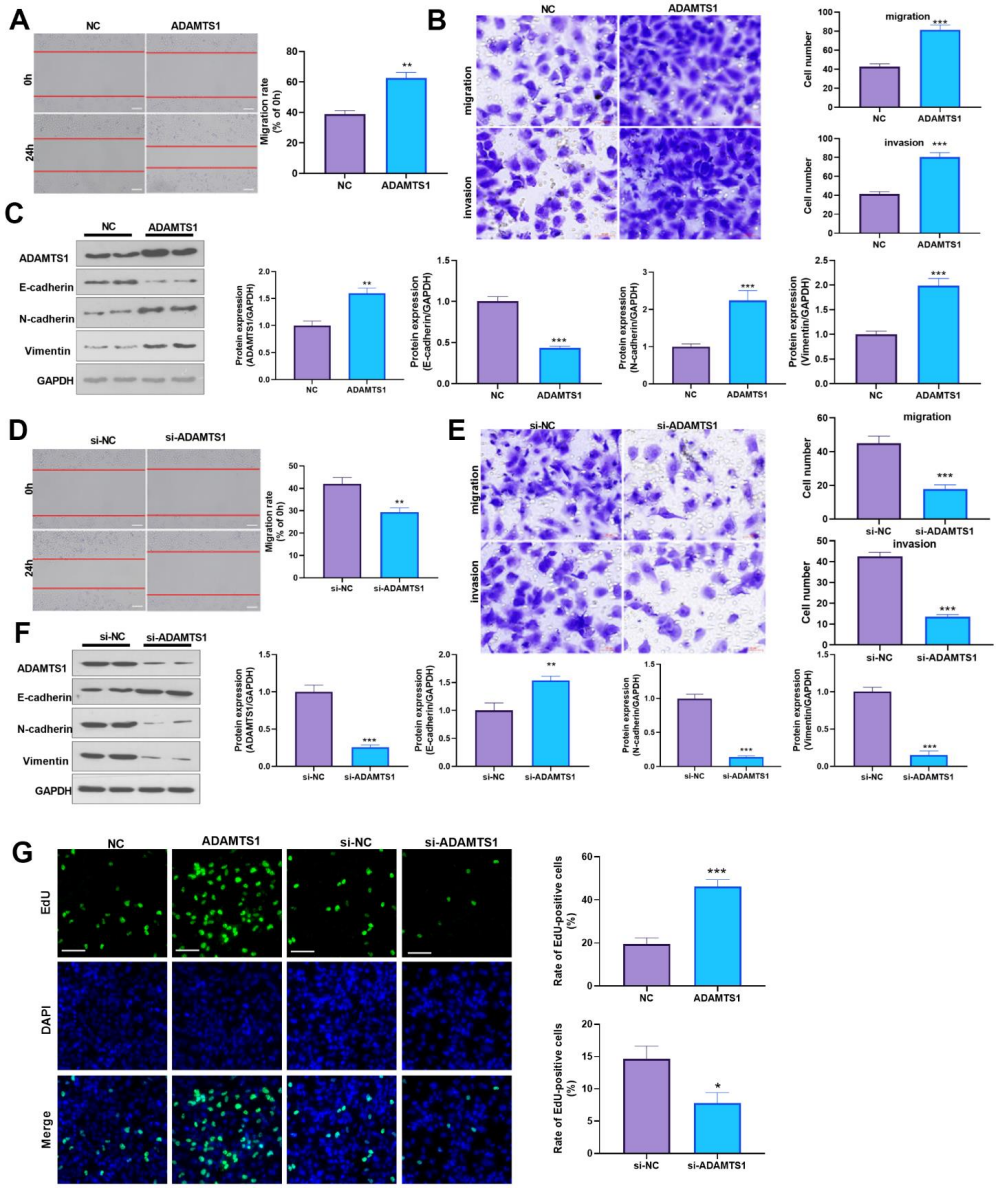
**ADAMTS1 promotes migration, invasion, and EMT of H226 cells.** ADAMTS1 overexpression plasmids or si-ADAMTS1 were transfected into H226 cells. (**A**) Wound-healing assay was used for detecting the migration of H226 cells transfected with ADAMTS1 overexpression plasmids. (**B**) Transwell examined the migration and invasion of H226 cells (scale bar=50 μm). (**C**) Protein expressions of E-cadherin, N-cadherin, and Vimentin in H226 cells were detected using Western blot. NC=negative control. (**D**) The wound-healing assay showed the migration of H226 cells after the knockdown of ADATMS1. (**E**) Transwell assays showed the migration and invasion of H226 cells after the knockdown of ADATMS1 (scale bar=50 μm). (**F**) Protein expressions of E-cadherin, N-cadherin, and Vimentin in H226 cells were detected using Western blot. (**G**) EdU staining was used for detecting cell proliferation. Scale bar=100 μm. *P<0.05, **P<0.01, ***P<0.001 compared with NC or si-NC group. N=3.

**Figure 4:**
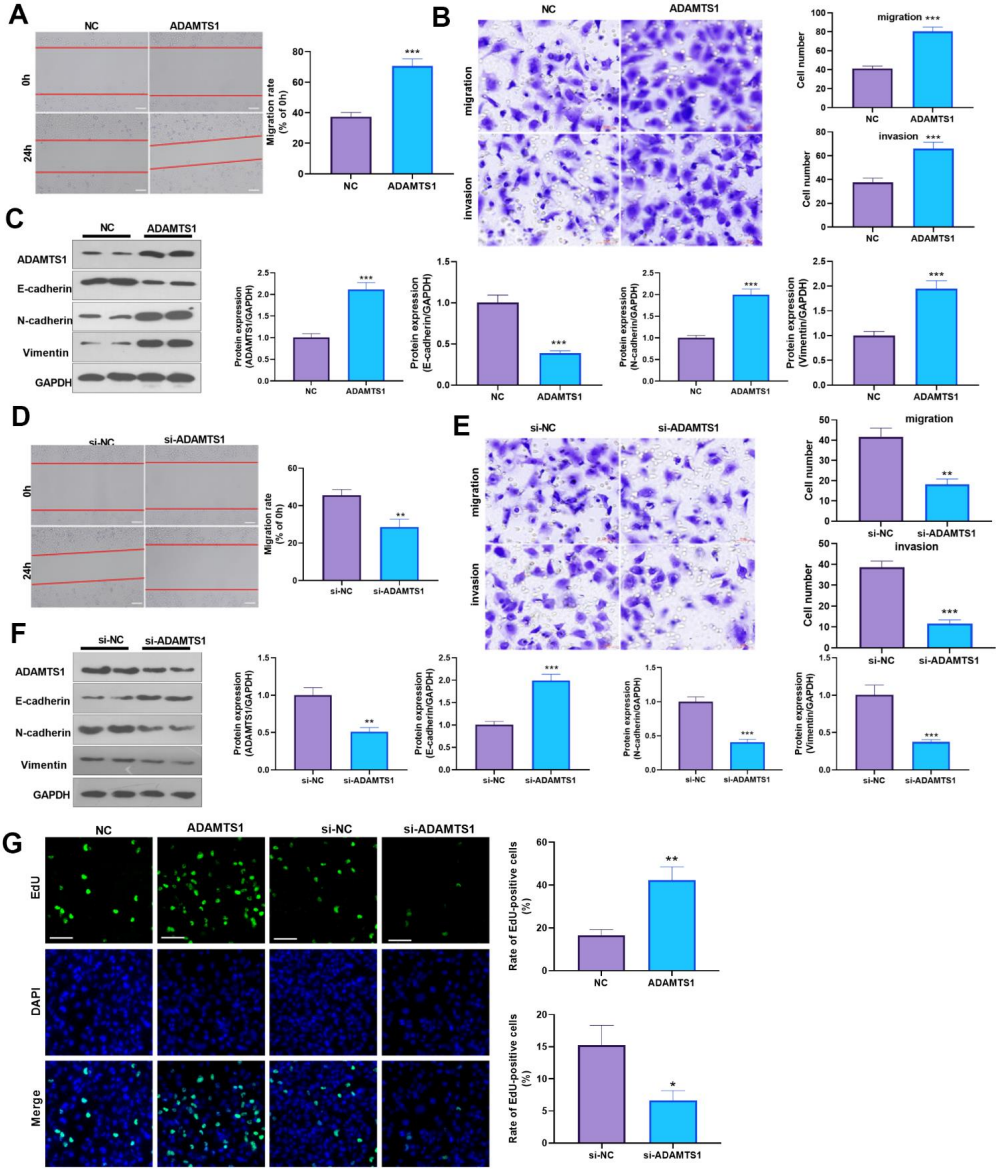
**ADAMTS1 promotes migration, invasion, and EMT of BEAS2B cells.** ADAMTS1 overexpression plasmids or si-ADAMTS1 was transfected into BEAS2B cells. (**A**) Wound-healing assay was used for detecting the migration of BEAS2B cells transfected with ADAMTS1 overexpression plasmids. (**B**) Transwell examined the migration and invasion of BEAS2B cells (scale bar=50 μm). (**C**) Protein expressions of E-cadherin, N-cadherin, and Vimentin in BEAS2B cells were detected using Western blot. NC=negative control. (**D**) The wound-healing assay showed the migration of BEAS2B cells after the knockdown of ADATMS1. (**E**) Transwell assays showed the migration and invasion of BEAS2B cells after the knockdown of ADATMS1 (scale bar=50 μm). (**F**) Protein expressions of E-cadherin, N-cadherin, and Vimentin in BEAS2B cells were detected using Western blot. (**G**) EdU staining was used for detecting cell proliferation. Scale bar=100 μm. *P<0.05, **P<0.01, ***P<0.001 compared with NC or si-NC group. N=3.

To determine the impact of ADAMTS1 on NSCLC cells, we measured the migrative ability of each group using a wound-healing assay, which showed migration of the A549 cells transfected with ADAMTS1 was significantly increased as compared with the control group (Figure 2A). The transwell assay results showed that compared with the control group, the migration and invasion cell count significantly increased after the up-regulation of ADAMTS1expression in A549 cells (Figure 2B). To investigate the effect of ADAMTS1 on the EMT of A549 cells, we used Western blot analysis to detect the expression of epithelial marker E-cadherin and interstitial cellular markers N-cadherin and vimentin. Our results showed that the protein expression of E-cadherin was significantly decreased after the upregulation of ADAMTS1 expression, whereas that of N-cadherin and vimentin was significantly increased (Figure 2C), suggesting that ADAMTS1 upregulation could promote the transformation of the A549 cells from an epithelial phenotype to a mesenchymal phenotype. We used siRNA to knock down the expression of ADATMS1. Wound-healing assay, transwell assay, and Western blot experiments showed si-ADAMTS1 inhibited the transformation of the A549 cells from an epithelial phenotype to a mesenchymal phenotype (Figure 2D–2F). Moreover, EdU staining showed that ADAMTS1 overexpression enhanced the proliferation of A549 cells, whereas ADAMTS1 knockdown had the reverse effect (Figure 2G). We also transfected ADAMTS1 or si-ADAMTS1 into the NSCLC cell line H226 and found that ADAMTS1 positively affected the migration, invasion, and EMT of H226 cells (Figure 3A–3G). We constructed ADAMTS1-overexpression or knockdown models in the human normal lung epithelial cell line BEAS2B cells. Interestingly, the migration, invasion, and EMT features were promoted when ADAMTS1 was overexpressed but reduced when ADAMTS1 was downregulated (Figure 4A–4G). These results suggest that ADAMTS1 promotes migration, invasion, and EMT in NSCLC cells and human normal lung epithelial cells.

### ADAMTS1 promotes lung metastasis of tumor cells in mice

**Figure 5:**
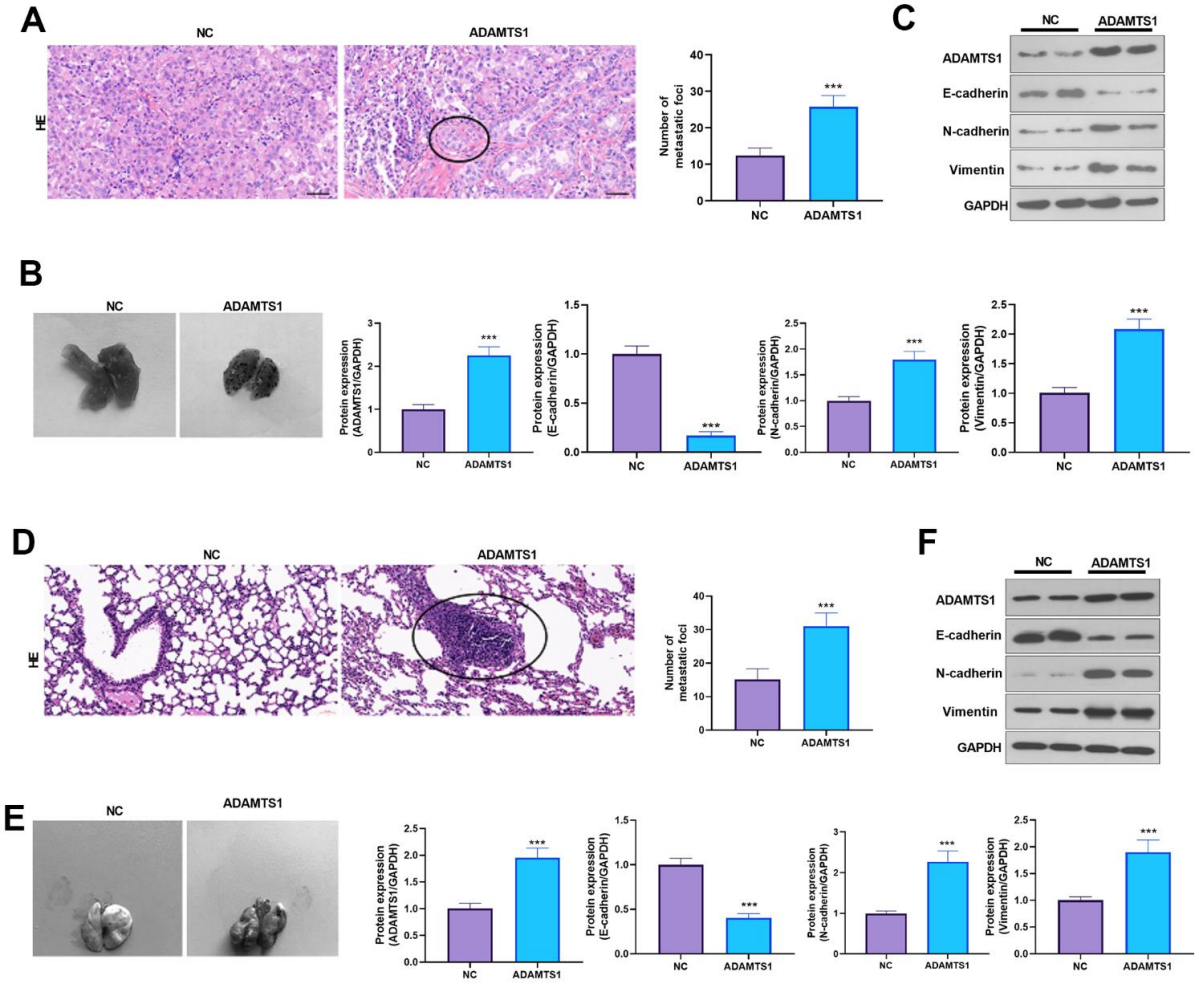
**ADAMTS1 promotes lung metastasis of tumor cells in mice.** A549 (**A**–**C**) and H226 cells (**D**–**F**) with ADAMTS1 upregulation were used for constructing an *in vivo* lung metastasis model in nude mice. (**A**) HE staining was used for detecting the pathological changes in lungs from mice injected with A549 cells. (**B**) The gross image of the lung. (**C**) Protein expression of E-cadherin, N-cadherin, and Vimentin in the lung tissues of nude mice was detected using Western blot. (**D**) HE staining was used for detecting the pathological changes in the lung from mice injected with H226 cells. (**E**) The gross image of the lung. (**F**) Protein expression of E-cadherin, N-cadherin, and Vimentin in the lung tissues of nude mice was detected using Western blot. ***P<0.001 compared with the NC group. N=3.

The effect of ADAMTS1 on the lung metastasis of NSCLC cells (A549 and H226 cells) was further investigated *in vivo*. HE staining and photographic results showed that overexpression of ADAMTS1 promoted the lung metastasis of tumor cells (Figure 5A, 5B, 5D, 5E). The Western blot analysis results showed that when compared with the control group, after transfection with ADAMTS1, the protein expression of E-cadherin in the lung tissue of the mice was reduced significantly (Figure 5C, 5F). The results suggested that ADAMTS1 promoted lung metastasis of NSCLC cells in mice and facilitated EMT.

### ADAMTS1 positively regulates the expression of TGF-β

**Figure 6:**
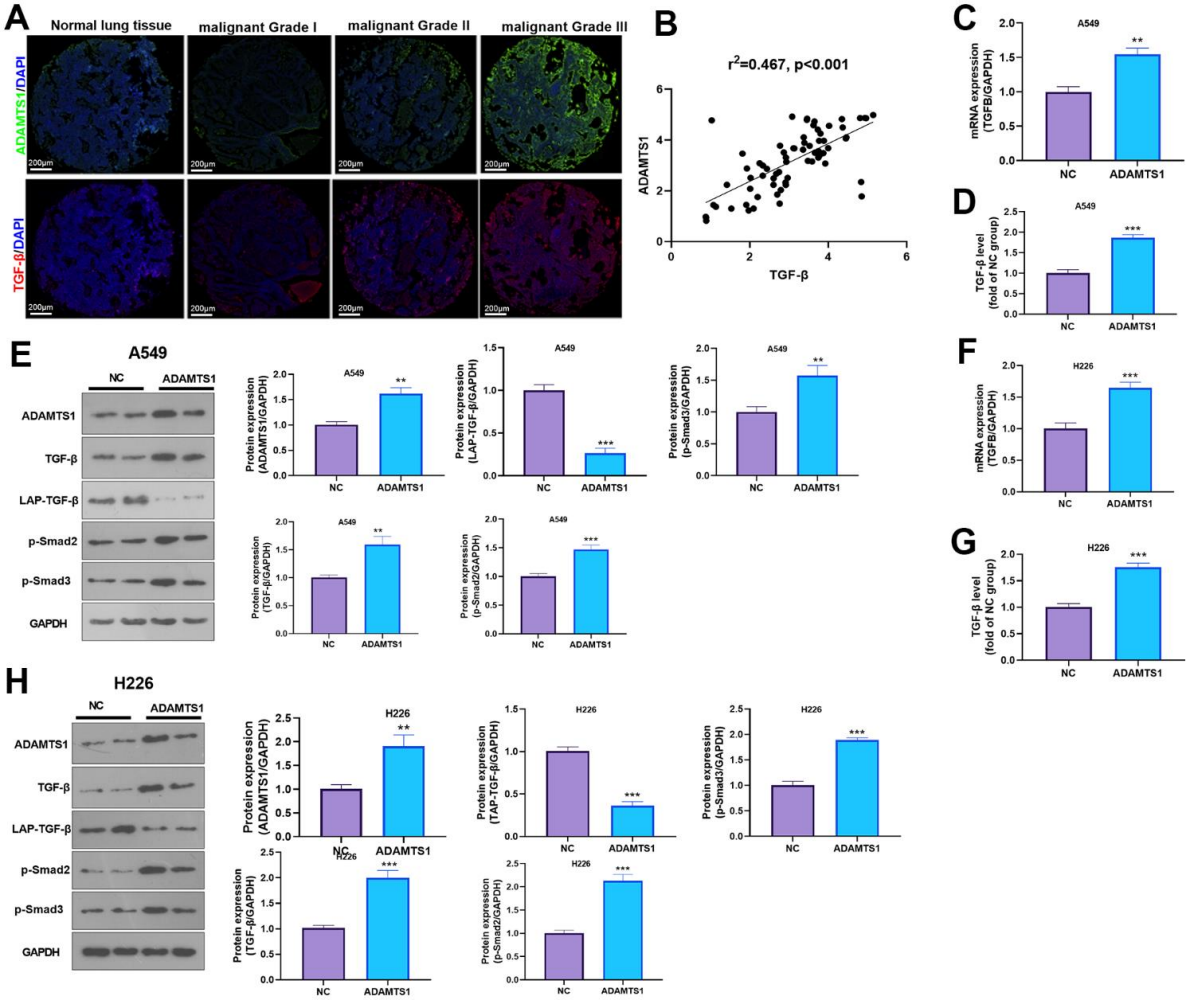
**ADAMTS1 positively regulates the expression of TGF-β in NSCLC cells or tumor tissues.** (**A**) Immunofluorescence was used for detecting ADAMTS16 and TGF-β in NSCLC tissues from different grades. Scale bar=200 μm. (**B**) qRT-PCR showed the mRNA expression level of TGF-β in lung tissues and adjacent normal tissues. (**C**) qRT-PCR showed the mRNA expression level of TGF-β in A549 cells overexpressing ADAMTS1. (**D**) Secretion of TGF-β protein in A549 cells detected using ELISA. (**E**) Protein expressions of TGF-β, LAP-TGF-β, and p-Smad2/3 in A549 cells were detected using Western blot. (**F**) qRT-PCR showed the mRNA expression level of TGF-β in H226 cells overexpressing ADAMTS1. (**G**) Secretion of TGF-β protein in H226 cells detected using ELISA. (**H**) Protein expressions of TGF-β, LAP-TGF-β, and p-Smad2/3in H226 cells were detected using Western blot. **P<0.01, ***P<0.001 compared with NC group. N=3.

The expression of ADAMTS1 and TGF-β was detected in NSCLC tissues and adjacent normal tissues using immunofluorescence and qRT-PCR. We found that the expression of TGF-β and ADAMST1 were both enhanced in NSCLC tissues (Figure 6A), and a positive relationship between ADAMTS1 and TGF-β was found (Figure 6B). qRT-PCR was used to detect the expression of TGF-β in lung cancer cell line A549 after transfection of ADAMTS1. The mRNA expression level of TGF-β in A549 cells overexpressing ADAMTS1 was significantly higher than that in the control group (Figure 6C). ELISA detection results also showed that up-regulation of ADAMTS1 can promote the secretion of TGF-β protein (Figure 6D). In addition, Western blot analysis results showed that compared with the control group, mature TGF-β protein expression and the phosphorylation of Smad2/3 in the A549 cells transfected with ADAMTS1 overexpressing plasmids were significantly increased (Figure 6E). In addition, we transfected ADAMTS1 overexpressing plasmids into H226 cells. It was found that ADAMTS1 upregulation led to enhanced TGF-β-Smad2/3 expression in H226 cells (Figure 6F–6H). Therefore, these data further implied that ADAMTS1 positively regulates the expression of TGF-β in NSCLC cells or tumor tissues.

### Effect of ADAMTS1 and TGF-β in NSCLC cells

**Figure 7:**
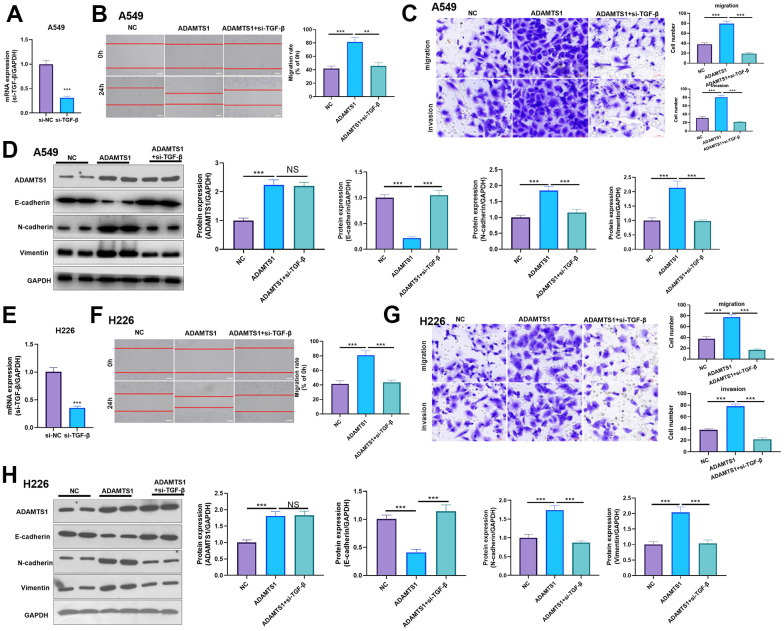
**Effects of ADAMTS1 and si-TGF-β on proliferation, cycle, migration, invasion, and EMT of the NSCLC cell line A549 and H226.** (**A**) qPCR showed that TGF-β was significantly reduced after transfecting siTGF-β into A549 cells. (**B**) The wound-healing assay showed migration of A549 cells co-transfected with ADAMTS1 and siTGF-β was significantly lower than that of cells transfected with ADAMTS1 (**C**) Transwell assay was used to detect migration and invasion of A549 cells (scale bar=50 μm). (**D**) Protein expressions of E-cadherin, N-cadherin, and Vimentin in A549 cells were detected using Western blot. (**E**) qPCR showed that TGF-β was significantly reduced after transfecting siTGF-β into H226 cells. (**F**) The wound-healing assay showed migration of H226 cells co-transfected with ADAMTS1 and siTGF-β was significantly lower than that of cells transfected with ADAMTS1. (**G**) Transwell assay was used to detect migration and invasion of H226 cells (scale bar=50 μm). (**H**) Protein expressions of E-cadherin, N-cadherin, and Vimentin in H226 cells were detected using Western blot. NS *P*>0.05, **P<0.01, ***P<0.001. N=3.

**Figure 8:**
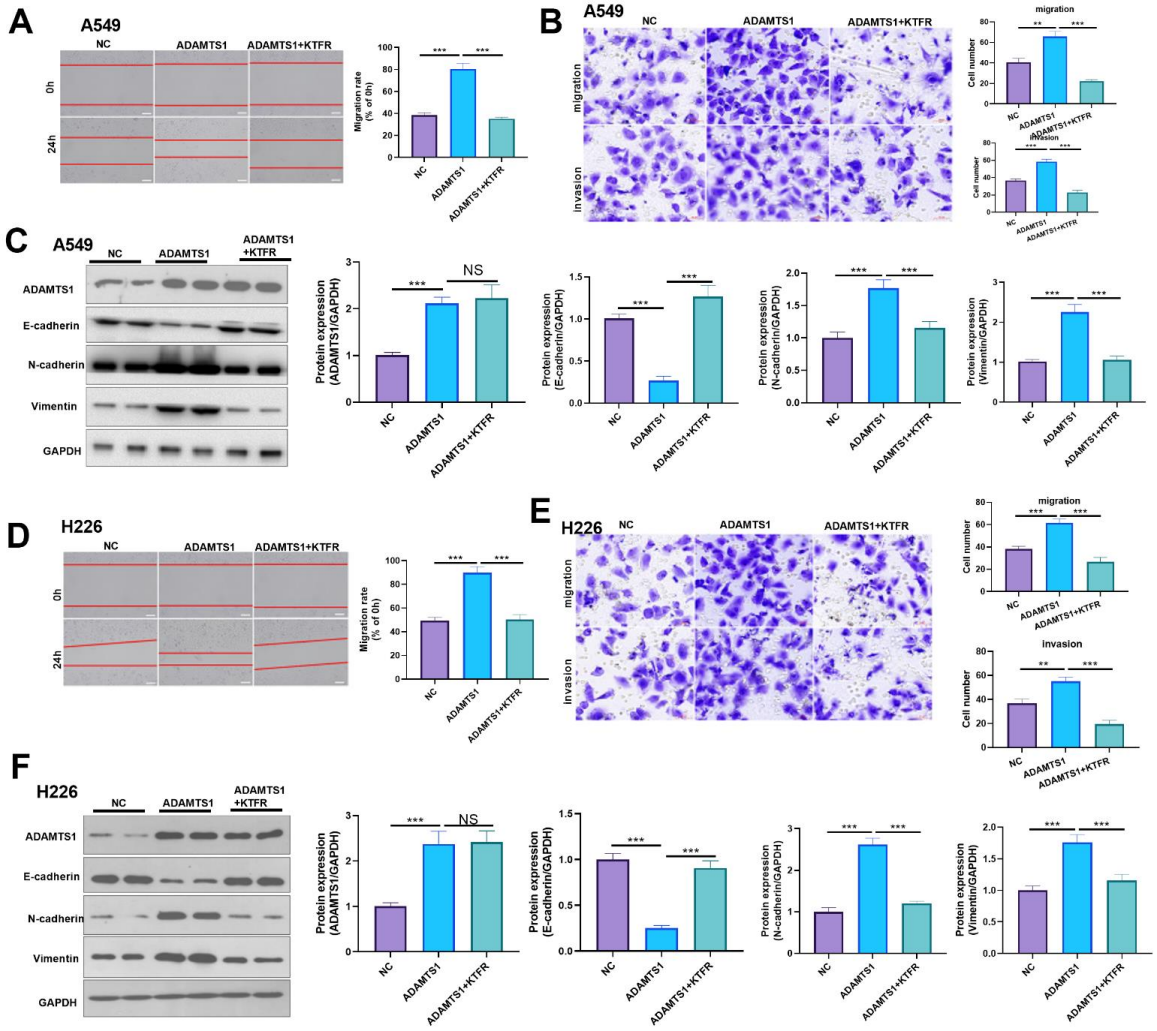
**Effects of ADAMTS1 and KTFR on proliferation, cycle, migration, invasion, and EMT of the NSCLC A549 and H226 cell line.** (**A**) The wound-healing assay showed the migration of A549 cells after treatment with KTFR. (**B**) Protein expressions of E-cadherin, N-cadherin, and Vimentin in A549 cells were detected using Western blot. (**C**) Transwell assay was used to detect migration and invasion of A549 cells (scale bar=50 μm). (**D**) The wound-healing assay showed the migration of H226 cells after adding KTFR. (**E**) Protein expressions of E-cadherin, N-cadherin, and vimentin in H226 cells were detected using Western blot. (**F**) Transwell assays were used to detect migration and invasion of H226 cells (scale bar=50 μm). NS *P*>0.05, **P<0.01, ***P<0.001. N=3.

**Figure 9:**
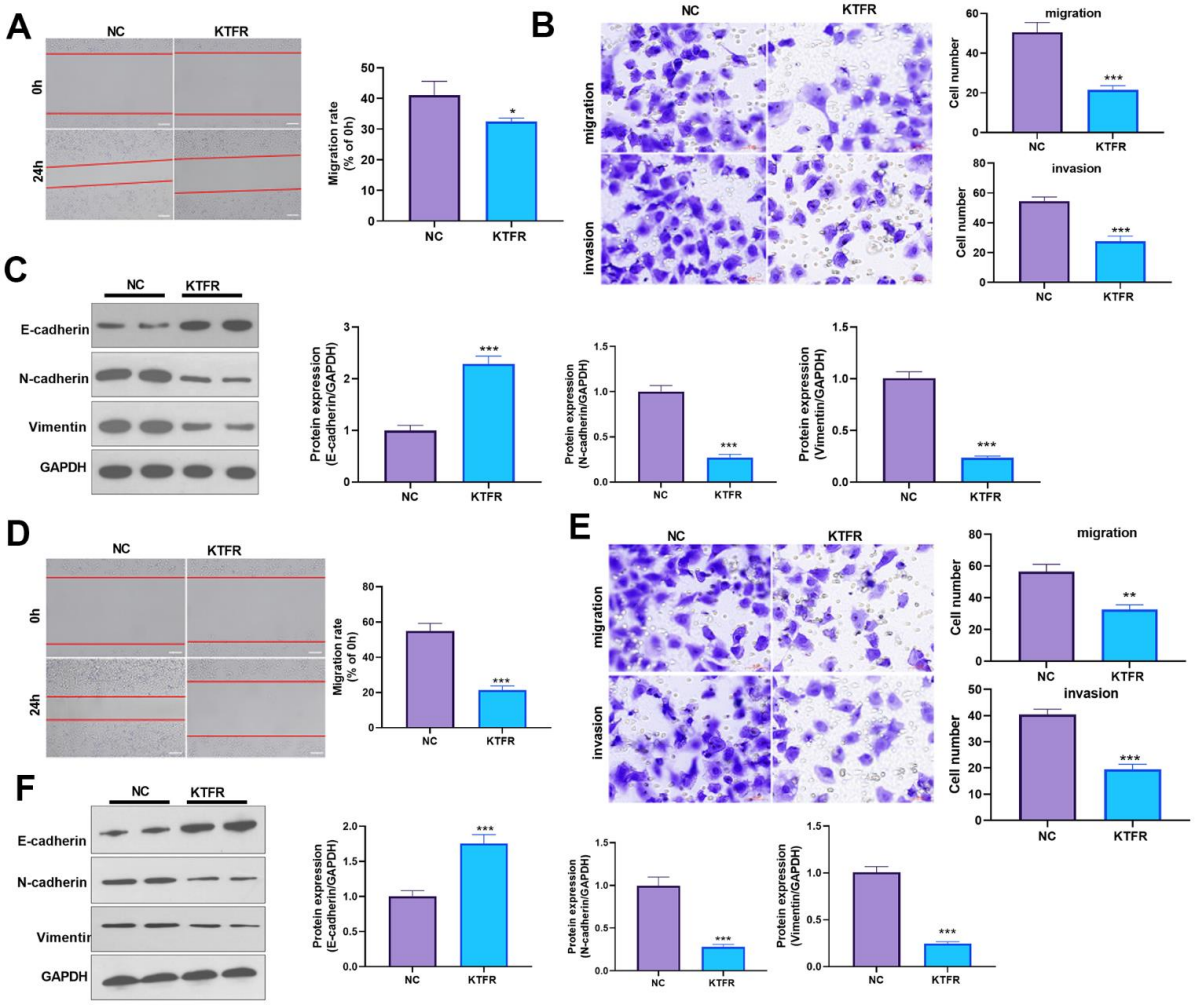
**Effects of KTFP on migration, invasion, and EMT of the NSCLC A549 and H226 cell line.** (**A**) Wound-healing showed the migration of A549 cells after adding KTFR. (**B**) Transwell assays showed the migration and invasion of A549 cells decreased after adding KTFR (scale bar=50 μm). (**C**) Protein expressions of E-cadherin, N-cadherin, and Vimentin in A549 cells were detected using Western blot analysis. (**D**) Wound-healing showed the migration of H226 cells was decreased after adding KTFR. (**E**) Transwell assays showed the migration and invasion of H226 cells were decreased after adding KTFR (scale bar=50 μm). (**F**) Protein expressions of E-cadherin, N-cadherin, and Vimentin in H226 cells were detected using Western blot. *P<0.05, **P<0.01, ***P<0.001 compared with NC group. N=3.

For confirming the downstream mechanism of TGF-β in ADAMTS1-mediated promotive effects in NSCLC cells, A549 cells and H226 cells were transfected with ADAMTS1 or co-transfected with ADAMTS1 and si-TGF-β. TGF-β was downregulated after si-TGF transfection (Figure 7A, 7E). The migration and invasion of A549 cells and H226 cells were detected by wound-healing assay or transwell assay. The results showed that migration of cells co-transfected with ADAMTS1 and si-TGF-β was significantly lower than that of cells transfected with ADAMTS1 (Figure 7B, 7C, 7F, 7G). Western blot showed that the protein expression of E-cadherin in A549 cells and H226 cells in ADAMTS1+siTGF-β group was significantly increased, while the protein expression of N-cadherin and Vimentin was significantly reduced (compared with ADAMTS1 group, Figure 7D, 7H). Furthermore, we used KTFR to stimulate A549 cells and H226 cells with high ADAMTS1 expression to inhibit the activation of TGF-β, and repeated the above experiment. The results showed that inhibition of TGF-β activation by KTFR could reverse the effect of ADAMTS1 on the phenotype of NSCLC cells (Figure 8A–8F). Furthermore, we used KTFR to stimulate A549 cells and H226 cells, and repeated the above experiment. The results showed that KTFR could inhibit NSCLC cells migration, invasion and EMT (Figure 9A–9F). Collectively, these results suggest that ADAMTS1 promotes the activation of TGF-β through the KTFR sequence and promoting lung cancer cell migration, invasion and EMT.

## DISCUSSION

In our study, we found that the expression of ADAMTS1 in NSCLC tumor tissues was significantly increased when compared with adjacent normal tissues, suggesting that the abnormal expression of ADAMTS1 correlates with NSCLC. Furthermore, using a series of cell experiments, we tested the effects of ADAMTS1 and TGF-β on the biological behavior of NSCLC cells. Our results showed that overexpression of ADAMTS1 could promote the proliferation, migration, and invasion of lung cancer cells. Furthermore, ADAMTS1 may be involved in the malignant behaviors of lung cancer cells by regulating the expression of TGF-β, suggesting that ADAMTS1 was involved in the regulation of NSCLC cells by positively regulating TGF-β.

Many studies have shown that the ADAMTS family plays a significant role in cell proliferation and apoptosis and in the occurrence, development, and metastasis of tumors [23–25]. ADAMTS1, a member of the ADAMTS family, plays an anti-angiogenic role by regulating vascular endothelial growth factor (VEGF) or fibroblast growth factor 2(FGF2) [26, 27]. In recent years, it has been reported that the expression of ADAMTS1 in pancreatic carcinoma tissues was significantly lower than that in adjacent normal tissues, while lymph node metastasis and retroperitoneal invasion in pancreatic carcinoma patients with higher ADAMTS1 expression were significantly increased [28]. Some studies have also found that the expression of ADAMTS1 decreased in colorectal carcinoma [29], prostate carcinoma [30], and other tumors, but increased in cervical carcinoma [31]. It has also been reported that overexpression of ADAMTS1 promotes pulmonary metastasis of TA3 mammary carcinoma and Lewis lung carcinoma cells [32]. The above results indicate that the role of ADAMTS1 is complex and differs in tumors of different origins. In addition to ADAMTS1, several ADAMTS members are found altered in lung cancer tissues. For example, ADAMTS5 was upregulated in NSCLC tissues and enhances the migration and invasion of lung cancer cells [33]. ADAMTS8 is an unfavorable predictor of lung cancer patients, and it mediates invasion and metastasis when expressed at a low level [34]. In higher stages and advanced NSCLC patients, enhanced VWF/ADAMTS-13 ratio and downregulated ADAMTS-13 were observed. And they were also associated with the final status of patients with lung cancer [35]. Those studies suggest the vital roles of ADAMTS members in lung cancer development.

EMT occurs due to the loss of cell polarity induced by epithelial cells, resulting in a loss of connection with the extracellular matrix and other epithelial cells and the acquisition of a capability to migrate and invade, and for anti-apoptosis [36, 37]. EMT is known to play a significant role in embryonic development and tissue remodeling, as well as in cancer and other diseases [38–40]. In our study, Western blot analysis showed that after over-expression of ADAMTS1 in NSCLC cell lines A549 and H226, the expression of the epithelial marker E-cadherin protein was significantly reduced, while that of the mesenchymal marker N-cadherin and Vimentin protein was significantly increased, indicating that ADAMTS1 could promote the transformation of NSCLC cells from an epithelial cell phenotype to a mesenchymal cell phenotype.

According to previous studies, there are several signaling pathways that are regulated by ADAMTS1, including HGF/c-MET signaling [41], VEGFC/VEGFR3 signal transduction pathway [42], and PI3K/Akt-eNOS/VEGF axis [43]. The TGF-β signaling pathway controls a variety of cellular processes, including cell growth, differentiation, and apoptosis, for example, and has been shown to play a complex role in inhibiting or promoting tumors in the occurrence of cancer [44, 45]. The inactivation or abnormal activation of TGF-β can lead to a variety of pathological states including malignant tumors, fibrotic diseases, and abnormal immune responses [46, 47]. TGF-β has been reported to be a typical inducer of EMT and a key factor in maintaining EMT in a variety of epithelial or epithelioid cancer cells [48]. In the early stage of cancer, TGF-β plays an antitumor role by inducing cytostasis and the apoptosis of normal and premalignant cells [49]. However, TGF-β exerts promotive effects in cancers by enhancing epithelial-mesenchymal transition (EMT) and metastasis of cancer cells [50]. Enhanced chemotherapy resistance was also found in TGF-β-overexpressed cells [51]. Moreover, TGF-β can also affect many signaling pathways in cells, including HGF-c-MET signaling [52], VEGF signaling [53] and PI3K/AKT pathway [54]. Interestingly, HGF/c-Met Axis [55], VEGF signaling [56], and PI3K/AKT pathway [57] are all involved in the proliferation, growth, and metastasis of lung cancer cells, and they are all identified as effective therapeutic targets in lung cancer. In this study, our findings showed that ADAMTS1 promoted the mRNA, and protein expression as well as the release of TGF-β in NSCLC cells, suggesting that ADAMTS1 positively regulates the expression level of TGF-β. We further transfected siTGF-β into NSCLC cells over-expressing ADAMTS1 and found that inhibiting the expression of TGF-β reversed the promotion of ADAMTS1 on NSCLC cell migration, invasion, and EMT. These data demonstrated that ADAMTS1 is involved in the regulation of NSCLC cells through regulating TGF-β expression.

One of the main characteristics of ADAMTS1 involves the presence of 3 kinds of TSP1 [58, 59]. Near the proximal TSP1 sequence, there is a KTFR motif consistent with the active KRFK sequence of TSP1 [60], which is involved in the interaction with TGF-β [61, 62]. KTFR, a short peptide on the ADAMTS1 protein, has been reported to be the key factor in ADAMTS1 activation of TGF-β [63], while KTFR alone can inhibit the activation of TGF-β [64]. We used KTFR to stimulate NSCLC cells with overexpression of ADAMTS1 and found that inhibition of TGF-β activation by KTFR could reverse the effects of ADAMTS1 on the biological behavior and EMT of NSCLC cells. Furthermore, this finding demonstrated that ADAMTS1 plays a role in NSCLC by regulating TGF-β. Moreover, we observed the effect of ADAMTS1 on NSCLC cells *in vivo* by constructing a tumor metastasis model in nude mice with NSCLC. Our results showed that the overexpression of ADAMTS1 could promote the metastasis of NSCLC cells to the lungs and the EMT, which was consistent with the *in vitro* results.

In conclusion, this study clarified that ADAMTS1 which was highly expressed in NSCLC cells and tissues could promote migration, invasion, and EMT. Furthermore, our study results showed that ADAMTS1 plays a complex regulatory role in the pathological process of NSCLC by positively regulating TGF-β.

## MATERIALS AND METHODS

### Research sample collection

We selected samples of lung cancer tissue and normal tissue adjacent to cancer (>5 cm from the tumor margin) from 69 patients (42 males, 27 females; mean age, 57.4 ± 9.4 years) who had undergone NSCLC treatment at our hospital, from December 2017 to November 2018. None of the patients had received pre-surgical chemotherapy, and all fresh samples were rapidly frozen in liquid nitrogen after isolation and confirmed according to postoperative pathology. All patients provided consent for the collection of their tissue samples, and the experiments were approved by the Ethics Committee of the Ningbo Municipal Hospital of Traditional Chinese Medicine in China (Approval No.:2018LL013).

### Cell culture and transfection

NSCLC cell lines (A549 or H226) and human normal lung epithelial cell line BEAS2B cells were obtained from American Type Culture Collection (ATCC, USA). As per supplier’s guidelines, the cells were maintained in a Roswell Park Memorial Institute 1640 (RPMI 1640) medium supplemented with 10% fetal bovine serum (both Thermo Fisher Scientific, Inc.) in a humidified water-jacketed incubator with 5% CO_2_ at 37° C. Every 2–3 days, according to the 1:3 passage, the A549 cells in the log phase were placed onto a cell culture plate. The cells were passaged in the laboratory for fewer than 6 months, performing all cell characterizations. When the cells were sub-cultured at between 70% and 80% confluence, according to transfection reagent Lipofectamine™ 2000 (Life Technologies, Carlsbad, CA, USA) specifications, the over-expression of ADAMTS1 plasmids PCDNA3.1-ADAMTS1 and control plasmids PCDNA3.1-vector were transfected into NSCLC cell lines (A549 or H226).

### qRT-PCR analysis

Total RNA was extracted using TRIzol reagent (Invitrogen Life Technologies, Carlsbad, CA, USA) according to the manufacturer's instructions, the content of the RNA was determined using UV spectrophotometry, and DNA ladder bands were detected using agarose gel electrophoresis. cDNA was synthesized using a RevertAid First Strand cDNA Synthesis Kit (Thermo Fisher Scientific, Inc.) according to the kit’s instructions, and internal reference gene GAPDH primers, ADAMTS1 primers, or TGF-β primers were added into the cDNA.

The primer sequences used were as follows:

GAPDH: F: 5’-CAAGGTCATCCATGACAACTTTG-3’

GAPDH: R: 5’-GTCCACCACCCTGTTGCTGTAG-3’

ADAMTS1: F: 5’-CTTGTGGCAGACCAGTCGAT-3’

ADAMTS1: R: 5’-TTCACCACCACCAGGCTAAC-3’

TGF-β: F: 5’-ATTCCTGGCGTTACCTTGG-3’

TGF-β: R: 5’-GCCCTGTATTCCGTCTCCTT-3’

The reaction conditions were as follows: pre-denaturation at 95° C for 10 min; at 95° C for 45 seconds, at 60° C for 45 seconds, at 72° C for 1 min, for a total of 35 cycles; then at 72° C for 10 min. Relative expression levels of mRNA and other indicators were calculated using the 2^-∆∆Ct^ method in triplicate. The amplification product was added into 2% agarose gel for electrophoresis, and the electrophoresis results were placed in the gel analysis system for semi-quantitative analysis of the bands. We calculated the qRT-PCR results using the 2^-∆∆Ct^ method.

### Western blot

Cell or tissue protein was extracted using RIPA Lysis Buffer and the protein concentration was determined in accordance with the BCA Protein Assays (Thermo Fisher Scientific, Inc.). The proteins (50 μg/well) were separated using 12% SDS-PAGE and transferred to a polyvinylidene fluoride (PVDF) membrane. The membranes were blocked with 5% non-fat dry milk powder for 1h at 37° C. Anti-ADAMTS1 antibody (1:500; ab45939; Abcam, Cambridge, UK), anti-TGF-β antibody (1:500; 3711S, Cell Signaling Technology, Inc), anti-E-cadherin (1:500; ab15148; Abcam), anti-N-cadherin antibody (1:500; ab76057; Abcam), anti-vimentin antibody (1:500; ab137321; Abcam), and internal reference GAPDH (1:1000; ab181602; Abcam) was added and incubated overnight at 4° C, after washing three times with Tris Buffered Saline Tween (TBST; Bio-Rad AbD Serotec Ltd). Horseradish peroxidase-labeled goat anti-rabbit secondary antibody (1:1000; ab150077; Abcam) was added and incubated at room temperature for 1h. An ECL chemiluminescence detection kit was used to visualize the protein bands.

### Immunohistochemical staining

Tissue specimens were dehydrated, transparentized, and immersed in wax after being fixed in 4% paraformaldehyde for 48 h. Using a paraffin slicing machine (Leica, RM2016, Wetzlar, Germany), the paraffin blocks were sliced into 5 μm serial paraffin sections, and the paraffin sections were placed in an oven at 65° C overnight, after which the paraffin sections were dewaxed and hydrated according to a gradient. Hydrated tissue sections were treated with 3% H_2_O_2_ and incubated at room temperature for 15 min to block endogenous peroxidase. The sections were placed in a citrate buffer and microwaved to repair the antigen. Then, 5% normal goat serum (Thermo Fisher Scientific, Inc.) was added and incubated at room temperature for 60 min. The primary antibodies, anti-ADAMTS11 antibody (1:500; ab45939; Abcam, Cambridge, UK) and anti-TGF-β antibody (1:500; 3711S, Cell Signaling Technology, Inc), were incubated with the sections at 4° C overnight. The secondary antibody, namely, horseradish peroxidase-labeled goat anti-rabbit secondary antibody (ab150077, 1:200, Abcam, Cambridge, UK) was incubated at room temperature for 30 min. Sections were obtained using a DAB horseradish peroxidase color development kit. Hematoxylin staining was performed at room temperature for 2 min, followed by dehydration and neutral resin packing. Positive images were observed with a vertical microscope and counted.

### HE staining

Paraffin sections were de-waxed in xylene and rehydrated using a graded alcohol series. Finally, the sections were counterstained with H&E (Beyotime Technology, Shanghai, China). The results were observed under a microscope and photographed.

### Transwell assay

After transfection, A549 cells were seeded on uncoated (for migration assays) and Matrigel-coated (for invasion assays) upper chambers. 100 uL cell suspension and 600 uL 10% serum medium (Thermo Fisher Scientific, Inc.) were added into the upper and lower chambers, respectively, and the cells were cultured in an incubator at 37° C at 5% CO_2_ for 24 h, followed by wiping of non-migrated or non-invaded cells. The chamber was fixed in 95% ethanol for 5 min and stained in 0.5% crystal violet for 10 min. The cells in the lower chamber were observed under a microscope.

### Enzyme-linked immunosorbent assay (ELISA)

PBS solution containing 1% fetal bovine serum (Thermo Fisher Scientific, Inc.) was used as a sealing solution with 350 μL/well and sealed at room temperature for 1 h. After sealing, we discarded the sealing solution, washed the plate, added 100 μL supernatant of cell culture medium to be tested, and incubated it at 25 ± 2° C for 2 h. After primary antibody incubation, the plates were washed and each well was incubated with horseradish peroxidase-labeled secondary antibody at room temperature for 2 h. After enzyme-labeled secondary antibody incubation, the plates were washed and a 100 μL TMB (Sigma-Aldrich) substrate solution was added to each well. After 30 min of color reaction at room temperature without light, 2 mol/L H_2_SO_4_ 50 μL was added to each well to terminate the reaction. The sample concentration was measured using an enzyme reader.

### EdU staining

Cell proliferation was tested using an EdU staining kit (Cat.No.40275ES60, Yeasen, Shanghai, China). NSCLC cell lines (A549 or H226) and human normal lung epithelial cell line BEAS2B cells were seeded in 24-well plates. Each well contained 1×10^5^ cells. The plates were put in an incubator with 5% CO_2_ at 37° C. After 24 hours, the cells were stained with EdU working solution for 2 hours. Next, the medium was removed. The cells were washed with PBS 2 times, fixed with 4% paraformaldehyde, and penetrated with 0.5% Triton X-100 in PBS. After that, the cells were reacted with 1× Click iTEdU reaction buffer, and the nucleus was stained by DAPI solution (Cat.No. 40728ES03, Yeasen, Shanghai, China). Finally, the EdU signal was observed using a fluorescence microscope (Olympus, Japan). The ratio of EdU-positive cells to DAPI-positive cells was counted.

### The tumor metastasis model in nude mice with NSCLC

BALB/c nude mice (n = 12; male; aged 4–6 weeks old; weight, 15–18 g) were kept at a constant room temperature of 22° C, 40-75% humidity, 12 h light/dark cycle, with free access to water and food. All nude mice were randomly divided into either a control group (n = 6) or an ADAMTS1 group (n = 6). After transfection, A549 cells were used to prepare a cell suspension with a cell density of 1×10^6^/ml. A 1 mL syringe was used to extract the cells and inject them subcutaneously into the axilla of the right hindlimb of nude mice (1×10^6^ cells, 0.2 ml in each mouse). After inoculation, the formation time and the growth status of subcutaneous transplanted tumors were observed regularly. After 6 weeks, all animals were sacrificed using cervical dislocation. The lungs were removed from the nude mice, fixed in a 4% paraformaldehyde fixative, and photographed, and the metastatic lesions in the lungs were observed. The experiment received the approval of the Animal Ethics Committee of Ningbo Municipal Hospital of Traditional Chinese Medicine (Approval No.:SYKK(Zhe)-2018-0012). All methods are reported in accordance with ARRIVE guidelines.

### Statistical analysis

All quantitative data for statistical analyses were derived from at least 3 independent experiments. Data are presented as mean ± standard deviation. A student’s t-test was used to compare the mean values between the 2 groups; an ANOVA test (Bonferroni post hoc test) was used to compare the mean among ≥3 groups. A p-value <0.05 was considered a statistically significant difference. All data and material in the manuscript are available.

### Availability of data and materials

The analyzed data sets generated during the study are available from the corresponding author upon reasonable request.
